# Variance and Entropy Assignment for Continuous-Time Stochastic Nonlinear Systems

**DOI:** 10.3390/e24010025

**Published:** 2021-12-24

**Authors:** Xiafei Tang, Yuyang Zhou, Yiqun Zou, Qichun Zhang

**Affiliations:** 1 Engineering Research Center of the Ministry of Education (Power Grid Security Monitoring and Control Technology), Changsha University of Science and Technology, Changsha 410114, China; xiafeitang@csust.edu.cn; 2 School of Engineering and The Built Environment, Edinburgh Napier University, Edinburgh EH11 4BN, UK; y.zhou@napier.ac.uk; 3 School of Automation, Central South University, Changsha 410083, China; yiqunzou@csu.edu.cn; 4 Department of Computer Science, University of Bradford, Bradford BD7 1DP, UK

**Keywords:** stochastic differential equation, Fokker–Planck–Kolmogorov equation, variance and entropy assignment

## Abstract

This paper investigates the randomness assignment problem for a class of continuous-time stochastic nonlinear systems, where variance and entropy are employed to describe the investigated systems. In particular, the system model is formulated by a stochastic differential equation. Due to the nonlinearities of the systems, the probability density functions of the system state and system output cannot be characterised as Gaussian even if the system is subjected to Brownian motion. To deal with the non-Gaussian randomness, we present a novel backstepping-based design approach to convert the stochastic nonlinear system to a linear stochastic process, thus the variance and entropy of the system variables can be formulated analytically by the solving Fokker–Planck–Kolmogorov equation. In this way, the design parameter of the backstepping procedure can be then obtained to achieve the variance and entropy assignment. In addition, the stability of the proposed design scheme can be guaranteed and the multi-variate case is also discussed. In order to validate the design approach, the simulation results are provided to show the effectiveness of the proposed algorithm.

## 1. Introduction

Stochastic systems, which are usually subjected to high levels of uncertainties and randomness, have become one of the major research fields due to their presence in the real-life systems. The randomness existing in the stochastic systems will result in bad control behaviour and lead to the instability of the controlled systems. Therefore, to enhance the performance of such systems, the influence of randomness has to be well controlled. To characterise the randomness of systems, variance has always been adopted as a statistical index when the randomness can be characterised as a Gaussian process, thus showing that the variance control method is regarded as an important design for stochastic systems analysis and implementation [1,2,3].

However, for the systems that cannot meet the Gaussian assumptions due to the nonlinearities, these results from the variance control method cannot be applied directly. It has been shown that the distributions of system variables can be twisted to non-Gaussian even if the noises in nonlinear systems are Gaussian noises. Based on the probability theory, variance is only the second moment which cannot reflect the full property of the non-Gaussian randomness [4]. To address that, the probability density function (PDF) control [5] becomes the solution for the stochastic nonlinear systems as PDF contains the full stochastic properties of random variables. The existing PDF control methods mostly focus on discrete-time systems. For the continuous-time stochastic nonlinear systems, it is difficult to find a solution analytically for the Fokker–Planck–Kolmogorov (FPK) equation [6]. Even though, entropy control [7] has been developed to overcome the non-Gaussian property and high-order moment problem of variance control, the analytical formulation of entropy for continuous-time dynamic system remains challenge since the FPK equation is hard to solve. Therefore, randomness control for continuous-time stochastic nonlinear systems is still regarded as a challenging topic.

We notice that the key factor for the randomness control is the system nonlinearity as the linear stochastic system can be handled by variance control. For linear stochastic systems, the entropy value is equivalent to variance. Motivated by Brownian motion [8,9], the randomness control for the stochastic nonlinear system can be achieved if the nonlinear process is able to be converted to linear process via the control design. Furthermore, the variance and entropy assignment can be achieved analytically to reflect the randomness. Following this idea, we consider a class of continuous-time stochastic nonlinear systems in this paper, where the backstepping design is adopted to stabilise the system variables. At the same time, the investigated nonlinear systems can be converted to linear format with designed parameter. The converted linear system structure can be analysed as an Ornstein–Uhlenbeck process [6], where the associated Fokker–Planck–Kolmogorov equation is solvable. In particular, the variance and entropy can be formulated analytically. Based on the formula, the designed parameters can be further obtained to achieve the randomness assignment.

Different from the stochastic distribution control [10], the randomness is investigated in a simplified approach and the analytical solution is obtained. Furthermore, the block backstepping design [11] and covariance assignment method [12] can be applied for multi-variate systems. In practice, the randomness assignment can also be used for filtering design [13], system identification and applications [14].

The rest of the paper is organised as follows. In Section 2, the formulation is given in terms of problem description and preliminaries related to the main results. Section 3 and Section 4 are the main contents of this paper, where the backstepping-based control law is proposed to stabilise the investigated stochastic system and the design parameter is analysed for variance assignment using Fokker–Planck–Kolmogorov equation. To validate the proposed control design, a numerical example is given in Section 5, where the results show that the variance can be assigned using the proposed control algorithm. The multi-variate system extension is discussed in Section 6 and the conclusions are given in Section 7 as the last part of this paper.

## 2. Formulation

In this paper, we first consider a univariate continuous-time stochastic affine nonlinear system which can be modelled by the following Itô process:

(1) $$\begin{array}{c} dx_{t}=\left(f\left(x_{t}\right)+v_{t}\right)dt+\sigma dW_{t} \end{array}$$

where $W_{t}$ stands for the Wiener process, $f\left(\cdot \right)$ stands for a smooth non-linear function, σ>0 denotes a real constant, $x_{t}$ and $v_{t}$ denote the system state and control input, respectively.

We can further re-write the investigated system model (1) by introducing an integrator while the following model can be obtained,

(2) $$\begin{array}{cc} dx_{t} & =\left(f\left(x_{t}\right)+v_{t}\right)dt+\sigma dW_{t}\\ dv_{t} & =u_{t}dt \end{array}$$

where $u_{t}$ denotes the new control signal while the control signal $v_{t}$ is the integral of $u_{t}$.

Then control objective can be described as designing $u_{t}$ such that $x_{t}$ is bounded in probability sense, then the variance and entropy of $x_{t}$ track the given desired values.

To achieve the system design, the preliminaries [15] are recalled as follows:

**Definition 1.**  *For any given $V\left(x_{t},t\right)\in C^{1,2}$$\left(R^{n}\times R_{+};R_{+}\right)$ associated with the stochastic differential Equation (1), the differential operator L is defined as follows:*

(3) $$\begin{array}{c} LV=\frac{\partial V}{\partial t}+\frac{\partial V}{\partial x_{t}}f\left(x_{t}\right)+\frac{\partial V}{\partial x_{t}}v_{t}+\frac{1}{2}Tr\left\{\frac{\partial^{2}V}{\partial x_{t}^{2}}\sigma^{2}\right\} \end{array}$$

**Lemma 1.**  *Consider the stochastic non-linear system model (1) and assume that $f\left(x_{t}\right)$ is $C^{1}$ in the arguments and $f\left(0\right)$ is bounded uniformly in t. If there exist functions $V\left(x_{t},t\right)\in C^{1,2}$$\left(R^{n}\times R_{+};R_{+}\right)$, $\mu_{1}\left(\cdot \right)$, $\mu_{2}\left(\cdot \right)$$\in K_{\infty }$, constants $c_{1}>0$, $c_{2}\geq 0$, and a nonnegative function $\bar{V}\left(x_{t},t\right)$, such that*

(4) $$\begin{array}{cc} \mu_{1}\left(|x_{t}|\right) & \leq V\left(x_{t},t\right)\leq \mu_{2}\left(|x_{t}|\right)\\ LV & \leq -c_{1}\bar{V}\left(x_{t},t\right)+c_{2} \end{array}$$

## 3. System Stabilisation

Based on the backstepping procedure [16], the virtual input signal can be designed as

(5) $$\begin{array}{c} \phi \left(x_{t}\right)=-f\left(x_{t}\right)-\theta x_{t} \end{array}$$

where θ>0 denotes the designed parameter for the controller.

In order to stabilise Equation (1), the virtual tracking error is formulated as

(6) $$\begin{array}{cc} z_{t} & =v_{t}-\phi \left(x_{t}\right)\\ \; & =v_{t}+f\left(x_{t}\right)+\theta x_{t} \end{array}$$

Substituting the error signal $z_{t}$ into Equation (32), we have

(7) $$\begin{array}{c} dx_{t}=\left(-\theta x_{t}+z_{t}\right)dt+\sigma dW_{t} \end{array}$$

Based upon Itô’s lemma, the following result is produced.

(8) $$\begin{array}{cc} dz_{t} & =dv_{t}-d\phi \left(x_{t}\right)\\ \; & =\left(u_{t}-\left(-\theta x_{t}+z_{t}\right)\frac{\partial \phi \left(x_{t}\right)}{\partial x}-\frac{\sigma^{2}}{2}\frac{\partial^{2}\phi \left(x_{t}\right)}{\partial x^{2}}\right)dt\\ & \;\;-\sigma \frac{\partial \phi \left(x_{t}\right)}{\partial x}dW_{t} \end{array}$$

One Lyapunov function candidate is selected to stabilise the investigated system model (32), which is given as follows.

(9) $$\begin{array}{c} V_{t}=V_{x}+V_{z}=\frac{1}{2}x_{t}^{2}+\frac{1}{4}z_{t}^{4} \end{array}$$

which results in

(10) $$\begin{array}{c} LV_{t}=LV_{x}+LV_{z} \end{array}$$

Using Definition 1, Lemma 1 and Young’s inequality, the following results can be evaluated as,

(11) $$\begin{array}{cc} LV_{x} & =x_{t}\left(-\theta x_{t}+z_{t}\right)+\frac{\sigma^{2}}{2}\\ \; & =-\theta x_{t}^{2}+x_{t}z_{t}+\frac{\sigma^{2}}{2}\\ \; & \leq -\theta x_{t}^{2}+\frac{1}{2}x_{t}^{2}+\frac{1}{2}z_{t}^{2}+\frac{\sigma^{2}}{2}\\ \; & =\left(-\theta +\frac{1}{2}\right)x_{t}^{2}+z_{t}^{2}+\frac{\sigma^{2}}{2}\\ \; & \leq \left(-\theta +\frac{1}{2}\right)x_{t}^{2}+\frac{1}{2}z_{t}^{4}+\frac{\sigma^{2}+1}{2} \end{array}$$

and

(12) $$\begin{array}{cc} LV_{z} & =z_{t}^{3}\left(u_{t}-\left(-\theta x_{t}+z_{t}\right)\frac{\partial \phi \left(x_{t}\right)}{\partial x}-\frac{\sigma^{2}}{2}\frac{\partial^{2}\phi \left(x_{t}\right)}{\partial x^{2}}\right)\\ \; & +\frac{3\sigma^{2}}{2}{\left(\frac{\partial \phi \left(x_{t}\right)}{\partial x}\right)}^{2}z_{t}^{2}\\ \; & \leq z_{t}^{3}\left(u_{t}+\theta x_{t}\frac{\partial \phi \left(x_{t}\right)}{\partial x}-z_{t}\frac{\partial \phi \left(x_{t}\right)}{\partial x}-\frac{\sigma^{2}}{2}\frac{\partial^{2}\phi \left(x_{t}\right)}{\partial x^{2}}\right)\\ \; & +\frac{1}{2}+\frac{9\sigma^{4}}{8}{\left(\frac{\partial \phi \left(x_{t}\right)}{\partial x}\right)}^{4}z_{t}^{4}\\ \; & =z_{t}^{3}\left(u_{t}+\theta x_{t}\frac{\partial \phi \left(x_{t}\right)}{\partial x}-\frac{\sigma^{2}}{2}\frac{\partial^{2}\phi \left(x_{t}\right)}{\partial x^{2}}\right)\\ \; & +\left(\frac{9\sigma^{4}}{8}{\left(\frac{\partial \phi \left(x_{t}\right)}{\partial x}\right)}^{4}-\frac{\partial \phi \left(x_{t}\right)}{\partial x}\right)z_{t}^{4}+\frac{1}{2} \end{array}$$

The control signal can be further developed as

(13) $$\begin{array}{c} u_{t}=-\theta x_{t}\frac{\partial \phi \left(x_{t}\right)}{\partial x}+\frac{\sigma^{2}}{2}\frac{\partial^{2}\phi \left(x_{t}\right)}{\partial x^{2}}-Cz_{t} \end{array}$$

where *C* stands for a designed real function.

Substituting the control signal into $LV_{z}$, Equation (10) can be written as

(14) $$\begin{array}{cc} LV_{t} & \leq \left(-\theta +\frac{1}{2}\right)x_{t}^{2}+\frac{1}{2}z_{t}^{4}+\frac{\sigma^{2}+2}{2}-Cz_{t}^{4}\\ \; & +\left(\frac{9\sigma^{4}}{8}{\left(\frac{\partial \phi \left(x_{t}\right)}{\partial x}\right)}^{4}-\frac{\partial \phi \left(x_{t}\right)}{\partial x}\right)z_{t}^{4}\\ \; & =\left(-\theta +\frac{1}{2}\right)x_{t}^{2}+\frac{\sigma^{2}+2}{2}\\ \; & +\left(\frac{1}{2}-C+\frac{9\sigma^{4}}{8}{\left(\frac{\partial \phi \left(x_{t}\right)}{\partial x}\right)}^{4}-\frac{\partial \phi \left(x_{t}\right)}{\partial x}\right)z_{t}^{4} \end{array}$$

The system state $x_{t}$ using $u_{t}$ is bounded in probability sense based on Lemma 1. Moreover, *C* can be further selected as follows to eliminate the $z^{4}$ nonlinear term.

(15) $$\begin{array}{c} C=\frac{1}{2}-\frac{9\sigma^{4}}{8}{\left(\frac{\partial \phi \left(x_{t}\right)}{\partial x}\right)}^{4}+\frac{\partial \phi \left(x_{t}\right)}{\partial x}-\kappa \end{array}$$

where κ≥0 denotes a free designed parameter.

As a result, we have

(16) $$\begin{array}{c} \frac{\partial \phi \left(x_{t}\right)}{\partial x}=-\frac{\partial f\left(x_{t}\right)}{\partial x}-\theta \end{array}$$

and

(17) $$\begin{array}{c} \frac{\partial^{2}\phi \left(x_{t}\right)}{\partial x^{2}}=-\frac{\partial^{2}f\left(x_{t}\right)}{\partial x^{2}} \end{array}$$

Substituting Equations (15)–(17) into the controller design (13), the complete control scheme can then be formulated as follows:

(18) $$\begin{array}{cc} u_{t} & =\theta x_{t}\left(\frac{\partial f\left(x_{t}\right)}{\partial x}+\theta \right)-\frac{\sigma^{2}}{2}\frac{\partial^{2}f\left(x_{t}\right)}{\partial x^{2}}\\ \; & -\left(\frac{1}{2}-\frac{9\sigma^{4}}{8}{\left(\frac{\partial \phi \left(x_{t}\right)}{\partial x}\right)}^{4}+\frac{\partial \phi \left(x_{t}\right)}{\partial x}-\kappa \right)\\ \; & \times \left(v_{t}+f\left(x_{t}\right)+\theta x_{t}\right) \end{array}$$

which leads to

(19) $$\begin{array}{cc} LV_{t} & \leq \left(-\theta +\frac{1}{2}\right)x_{t}^{2}-\kappa z_{t}^{4}+\frac{\sigma^{2}+2}{2} \end{array}$$

Thus, the closed-loop system with the designed parameter $\theta \geq \frac{1}{2}$ is bounded in probability sense.

## 4. Variance and Entropy Assignment

Substituting the control signal into $LV_{z}$ shows that the error signal $z_{t}$ is also bounded in the probability sense once the free parameter meets κ>0. Thus, the closed-loop system with the given control scheme can be further considered as the Ornstein–Uhlenbeck process.

(20) $$\begin{array}{c} dx_{t}=-\theta x_{t}dt+\sigma dW_{t} \end{array}$$

while the Fokker–Planck–Kolmogorov equation can be formulated as follows:

(21) $$\begin{array}{c} \frac{\partial p(x,t)}{\partial t}=\theta \frac{\partial }{\partial x}\left(xp(x,t)\right)+\frac{\sigma^{2}}{2}\frac{\partial^{2}p\left(x,t\right)}{\partial x^{2}} \end{array}$$

where p(x,t) denotes the PDF and *x* denotes the random variable of $x_{t}$.

Note that the associated FPK equation is a linear partial differential equation; the analytical solution can be formulated analytically as follows:

(22) $$\begin{array}{c} p\left(x,t\right)=\sqrt{\frac{\theta }{\pi \sigma^{2}\left(1-e^{-2t\theta }\right)}}\exp\left(-\frac{\theta }{\sigma^{2}}\frac{{\left(x-x_{0}e^{-t\theta }\right)}^{2}}{1-e^{-2t\theta )}}\right) \end{array}$$

where $x_{0}$ stands for the initial value of $x_{t}$ at $t_{0}$.

Since the solution of this FPK equation is a Gaussian distribution, the mean value and variance can be obtained analytically. It implies that the presented control scheme governs the non-Gaussian PDF of $x_{t}$ to re-shape as a Gaussian distribution. In particular, the formula of mean value and variance can be obtained as follows:

(23) $$\begin{array}{cc} E\left(x_{t}\right) & =x_{0}e^{-t\theta } \end{array}$$

(24) $$\begin{array}{cc} Var\left(x_{t}\right) & =\frac{\sigma^{2}}{2\theta }\left(1-e^{-2t\theta }\right) \end{array}$$

where $E\left(\cdot \right)$ and $Var\left(\cdot \right)$ stand for the operators of the mean value and the variance value. It has been shown that both mean value and variable can be adjusted via the designed parameter turning in the presented control scheme θ. In addition, the mean value reaches zero with θ>0 is considered as the convergence rate.

Denoting a desired variance function as $r\left(t\right)$, θ can then be further developed as a function of *t* such that the following equation holds.

(25) $$\begin{array}{c} \frac{\sigma^{2}}{2\theta }\left(1-e^{-2t\theta }\right)=r\left(t\right) \end{array}$$

Note that this equation can be rewritten as the following form,

(26) $$\begin{array}{c} e^{-2t\theta }=-\frac{2r\left(t\right)}{\sigma^{2}}\theta +1 \end{array}$$

Using the Lambert W function [17], the equation can be solved and the solution is given as follows:

(27) $$\begin{array}{c} \theta =\frac{W_{0}\left(-\frac{t\sigma^{2}}{r\left(t\right)}e^{-\frac{t\sigma^{2}}{r\left(t\right)}}\right)}{2t}+\frac{\sigma^{2}}{2r\left(t\right)} \end{array}$$

where $W_{0}\left(\cdot \right)$ is the Lambert W function. Note that $W_{0}\left(\cdot \right)$ can be adopted when $2r_{c}\theta \geq \sigma^{2}$.

To ensure the $W_{0}\left(\cdot \right)$ function can be used above, the stationary solution can be practically implemented if $r\left(t\right)=r_{c}$ is a real positive constant.

(28) $$\begin{array}{c} \theta_{s}=\lim_{t\to \infty }\theta =\frac{\sigma^{2}}{2r_{c}} \end{array}$$

Note that the solution meets the condition of $W_{0}\left(\cdot \right)$ where $2r_{c}\theta_{s}\geq \sigma^{2}$.

We can achieve the entropy assignment for the random variable $x_{t}$ as the variance value is correlated to Shannon’s entropy subjected to the linear stochastic process. Based on the definition, we have

(29) $$\begin{array}{c} H\left(x_{t}\right)=\frac{1}{2}\left(\log\left(2\pi Var\left(x_{t}\right)\right)+1\right) \end{array}$$

where $H\left(\cdot \right)$ stands for the Shannon’s entropy.

Based on the equation above, we can establish the link between the desired variance function and the pre-specified entropy function.

(30) $$\begin{array}{c} r\left(t\right)=\frac{1}{2\pi }e^{2H_{r}\left(t\right)-1} \end{array}$$

where $H_{r}\left(t\right)$ stands for the pre-specified desired entropy function.

Substituting Equation (30) into Equation (28), the parameter θ can be confirmed. Similar to the variance assignment, the stationary solution for entropy assignment is achieved if $H_{r}\left(t\right)=H_{c}$ is a real constant.

(31) $$\begin{array}{c} \theta_{s}=\pi \sigma^{2}e^{1-2H_{c}} \end{array}$$

As Shannon’s entropy is regarded as a special case of Rényi’s entropy, the presented control scheme can be extended to various entropies. As a result, the entropy optimisation can be further achieved for non-Gaussian filtering designs [13] and performance enhancement [18].

## 5. Simulation

The following numerical example is designed for validating the presented scheme. Matching the investigated system model, the parameters are confirmed as follows:

(32) $$\begin{array}{cc} dx_{t} & =\left(x_{t}^{3}+v_{t}\right)dt+0.2dW_{t}\\ dv_{t} & =u_{t}dt \end{array}$$

To show the assignment procedure, the reference variance value of $x_{t}$ is given as 0.02 while it leads to the parameter selection θ=1. In particular, $\theta =1>\frac{1}{2}$ implies that the designed system should be bounded in the probability sense. To start up the backstepping design, the virtual tracking error can be described by the following dynamic model:

(33) $$\begin{array}{cc} dz_{t} & =\left(u_{t}+\left(z_{t}-x_{t}\right)\left(3x_{t}^{3}+1\right)+\frac{0.04}{3}x_{t}\right)dt\\ \; & +0.2\left(3x_{t}^{2}-1\right)dW_{t} \end{array}$$

Thus, the procedure can be implemented for achieving the proposed control objective.

**Figure 1:**
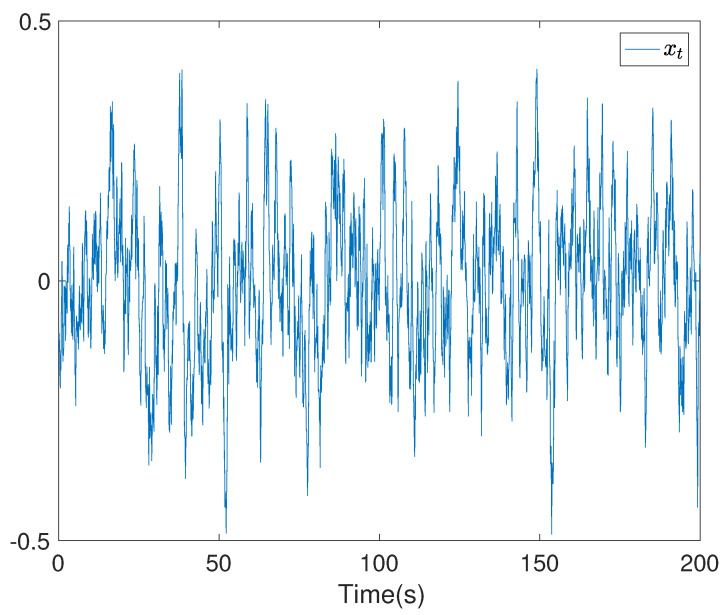
The trajectory of the system state $x_{t}$.

**Figure 2:**
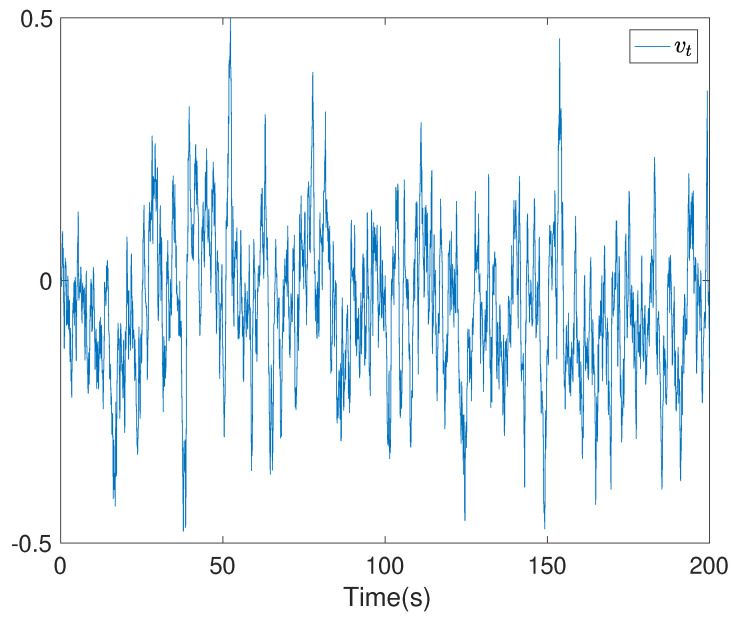
The control input with integrator $v_{t}$.

**Figure 3:**
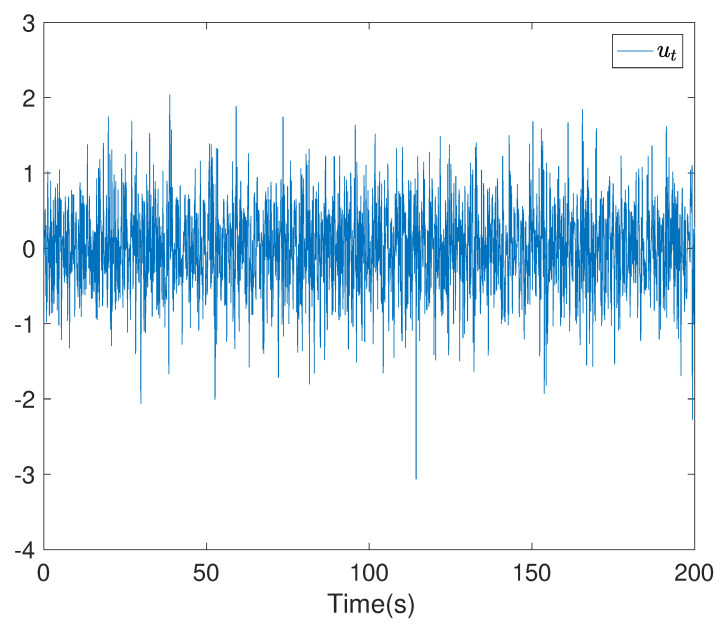
The control input without integrator $u_{t}$.

**Figure 4:**
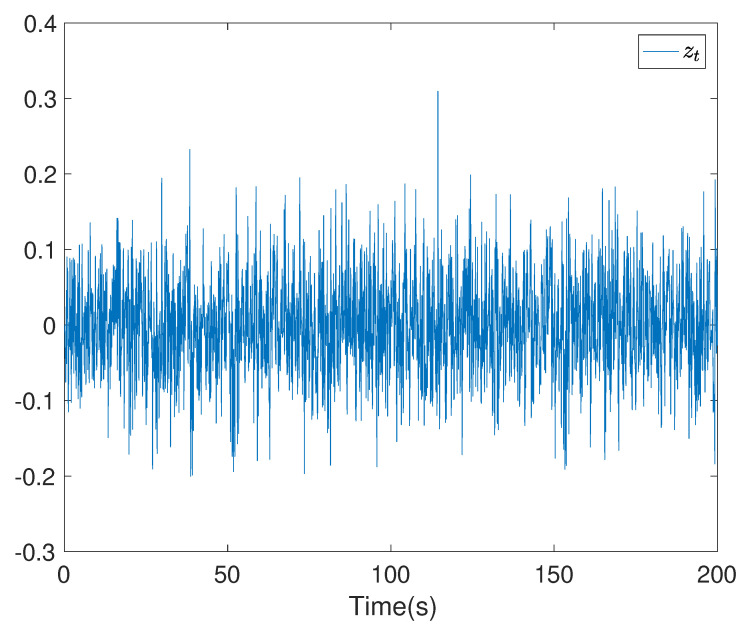
The trajectory of the virtual error $z_{t}$.

**Figure 5:**
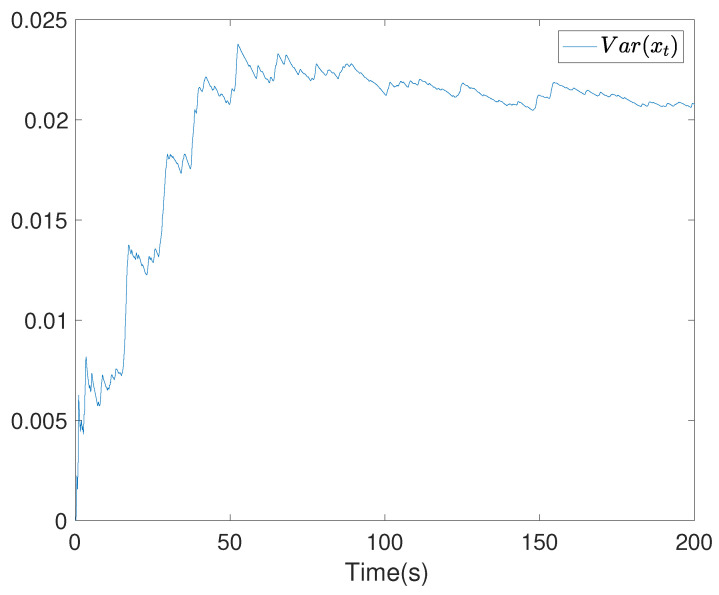
The variance value curve of the system state $x_{t}$.

**Figure 6:**
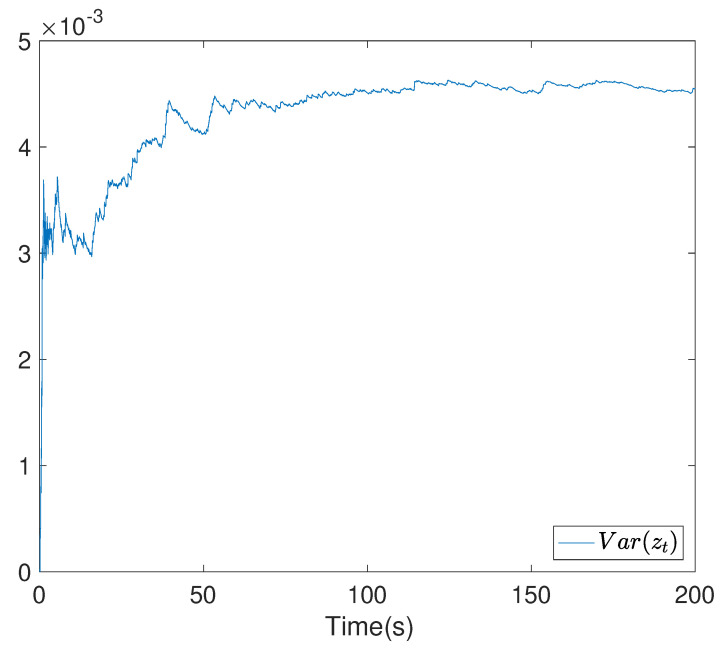
The variance value curve of the virtual error $z_{t}$.

**Figure 7:**
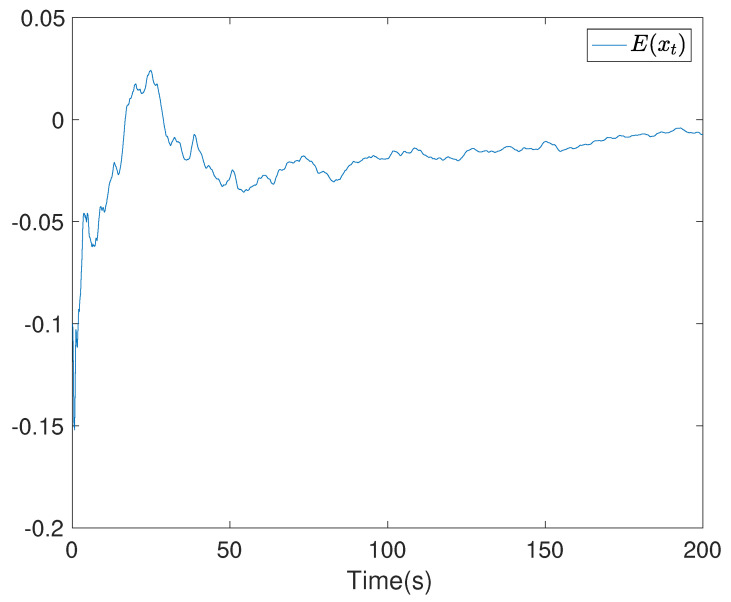
The mean value curve of the system state $x_{t}$.

**Figure 8:**
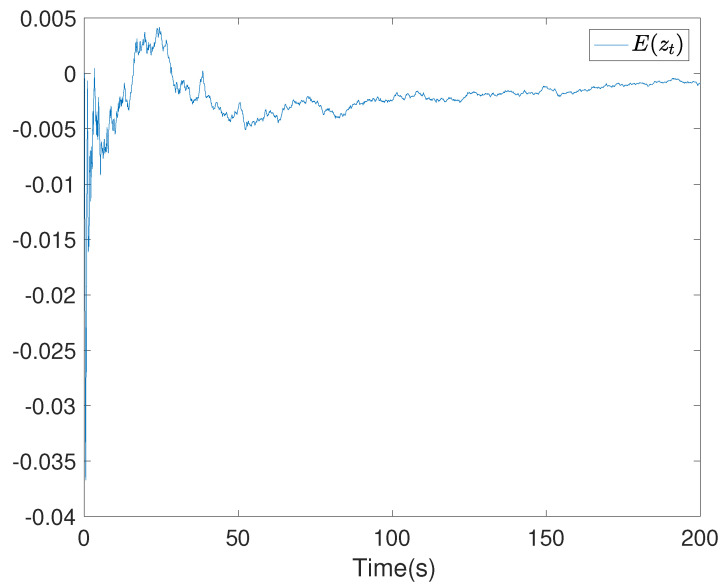
The mean value curve of the virtual error $z_{t}$.

To show the simulation results, we first discretise the given model using with the sampling time 0.1. Then the computational results are demonstrated in Figure 1, Figure 2, Figure 3, Figure 4, Figure 5, Figure 6, Figure 7 and Figure 8. In particular, the trajectory of the system state $x_{t}$ is shown in Figure 1, where the system variable stabilisation is achieved, while the control input signal with integrator $v_{t}$ and the signal without integrator $u_{t}$ are given in Figure 2 and Figure 3, respectively. Figure 4 shows the randomness attenuation where the virtual tracking error $z_{t}$ reaches zero. The variance values of $x_{t}$ and $z_{t}$ are indicted in Figure 5 and Figure 6, in which the variance value of $x_{t}$ converges to the assigned reference value and the transient error comes from the virtual tracking error stabilisation progress. The mean values of $x_{t}$ and $z_{t}$ are also given in Figure 7 and Figure 8, respectively. Note that both mean values are approaching zero. However, Lemma 2 implies that the error of the mean value of $z_{t}$ still exists due to the Lyapunov theorem-based analysis, where an arbitrary small non-zero error exists. As a result, the mean value and variance value of $x_{t}$ may be affected by the non-zero virtual tracking error, although it is arbitrarily small.

## 6. Discussion

In the system design above, only the univariate system was investigated to indicate the framework via backstepping. To extend this result, the main challenge comes from the multivariate backstepping design. As a solution, the block backstepping design is an implementable solution similar to the design procedure in [11]. In particular, we can consider the following extended system model with multi-dimensional system variables.

(34) $$\begin{array}{cc} d{\bar{x}}_{t} & =\bar{f}\left({\bar{x}}_{t}+{\bar{v}}_{t}\right)dt+\Sigma d{\bar{W}}_{t}\\ y_{t} & =Cx_{t} \end{array}$$

where $\bar{f}\left(\cdot \right)$ stands for a known smooth non-linear function $\bar{f}:R^{n\times 1}\to R^{n}$, ${\bar{W}}_{t}$ denotes n-dimensional Wiener process, Σ denotes a given as a real positive square matrix with n dimensions. ${\bar{x}}_{t}\in R^{n}$ and $y_{t}\in R^{1}$ stand for the system state vector and system output, respectively. ${\bar{v}}_{t}\in R^{n}$ denotes the control input. $C\in R^{n}$ denotes a vector-valued coefficient.

Following the presented design approach, the candidate of Lyapunov functions can be re-used where the vector-value variables will be used. Since Lemma 1 holds for multivariate system, the developed result in this paper can be extended directly following the block backstepping design. Notice that the linear Ornstein–Uhlenbeck process will be in the multi-dimensional form which leads to the difficulty of solving the FPK equation, as the joint probability density function has to be involved into the multivariate case. To avoid this problem, the design parameter θ should be selected as the positive diagonal matrix. Then a set of FPK equations can be obtained where the vector state can be decomposed as single variables. Therefore, the presented parameter selection scheme can also be re-used for multivariate systems. Alternatively, the converted linear multivariate systems can be further adjusted by covariance assignment which means an additional control signal will be introduced into the system design.

## 7. Conclusions and Perspectives

In this paper, a new randomness assignment framework is proposed for continuous-time stochastic nonlinear systems which are described by stochastic differential equations. The core idea is to convert the nonlinear system via control design, then the randomness can be fully characterised by variance. In particular, the backstepping procedure is first used to stabilise the variables of the investigated system with designed parameters. In this way, the converted linear system can be further described by the Ornstein–Uhlenbeck process. Thus, the associated Fokker–Planck–Kolmogorov equation is then analytically solvable which leads to the formula of variance and entropy value. Then, the assignment can be achieved by selecting designed parameter of backstepping. A numerical example is given as a validation of the presented method and a discussion is further given for the multivariate systems. In the future, the covariance control theory can be further merged into the presented framework to enhance the flexibility of the assignment for the multivariate cases.
